# Analysis of the differential expression and prognostic relationship of DEGs in AML based on TCGA database

**DOI:** 10.1186/s40001-023-01060-3

**Published:** 2023-02-27

**Authors:** Yue Gao, Yinnong Jia, Zhengmin Yu, Xinyu Ji, Xiaowen Liu, Lei Han, Hengdong Zhang, Baoli Zhu, Ming Xu

**Affiliations:** 1 Department of Occupational Disease Prevention, Jiangsu Provincial Center for Disease Control and Prevention, No. 172 Jiangsu Road, Nanjing, 210009 China; 2 Public Health Research Institute of Jiangsu Province, Nanjing, 210009 China; 3 School of Pharmaceutical Sciences and Yunnan Key Laboratory of Pharmacology for Natural Products, Kunming Medical University, Kunming, 650500 China; 4 Center for Global Health, School of Public Health, Nanjing Medical University, Nanjing, 211166 China

**Keywords:** Acute myeloid leukemia, TCGA, DEGs, Survival analysis

## Abstract

**Background:**

Acute myeloid leukemia (AML) is a common and lethal hematological malignant hyperplastic disease originating from hematopoietic stem cells. The purpose of this study is to obtain the key differentially expressed gene (DEG) related to the survival of AML by The Cancer Genome Atlas (TCGA) database and to verify these genes by a clinical follow-up investigation, in order to identify valuable predictive and prognostic biomarkers for early diagnosis of AML and predict the survival rates.

**Methods:**

The RNA sequencing (RNA-Seq) data and clinical information of TCGA-LAML were downloaded from the TCGA database. After that we (1) screened the survival-related DEGs by Cox regression analysis, (2) selected the cytogenetics risk-related DEGs by DESeq2 R package, and (3) filtrated the genes in the top10 pathways of up-regulated and down-regulated of Normalization Enrichment Score (NES) by Gene Set Enrichment Analysis (GSEA). Finally, we focused the intersectional genes of above three parts as the key gene of the present study. The following Multivariate.

## Introduction

Leukemia is a malignant disease of the hematopoietic system. Based on disease progression and cell types, leukemia can be classified as acute lymphocytic leukemia (ALL), chronic lymphocytic leukemia (CLL), acute myelogenous leukemia (AML), and chronic myelogenous leukemia (CML) [1]. AML is a group of malignant clonal diseases originating from bone marrow hematopoietic stem cells, characterized by abnormal differentiation of hematopoietic stem cells and excessive proliferation of myeloid progenitor cells [2]. The abnormal accumulation of leukemia cells leads to the inhibition of normal hematopoietic cell growth and the infiltration of leukemia cells in the bone marrow, resulting in multiple organ failures and symptoms, such as anemia, bleeding, and infection [3]. The onset of AML can cover all periods of lifetime, but it mostly occurs in the elder-age and male patients, the incidence of which usually increases following the age, with a median of 67 years at diagnosis [4, 5]. According to the previous study, approximate 80% of AML occurs in adults [6].

At present, with the development of new diagnosis and treatment technologies, the survival rate of AML patients has significantly improved, but the long-term survival rate of patients still remains poor. For patients  < 60 years old, according to the related previous studies, the 5-year overall survival (OS) rate is less than 40%; for the majority of patients with AML (aged over 60 years old), the 5-year OS rate is only 10–20% [7, 8]. Meanwhile, AML patients mainly have accompanied with poor prognosis. Even though most patients achieve a complete remission (CR) with intensive induction chemotherapy, over half of young adult patients and about 90% of elderly patients still succumb to the disease [9]. The culprits of above phenomenon could be regarded as the limited current knowledge of the underlying molecular mechanisms and its progression of AML, and the low-effective early clinical diagnosis. Some studies show that patients with different subtypes, different karyotypes, different gene expressions, and mutation types have different prognosis [10]. Therefore, finding out effective target genes and prognostic molecules is of great significance to the early diagnosis and prognosis of patients with AML.

With the developments of gene detecting and computing sciences, the high-throughput sequencing technologies and bioinformatics analyses have been widely used in clinical researches to screen meaningful oncogene and epigenetic changes. It is helpful to identify the differentially expressed genes (DEGs), functional pathways involved in AML carcinogenesis, and the biomarkers for diagnosis or prognosis. In order to conduct a comprehensive study of the human cancer genome, the United States initiated the establishment of The Cancer Genome Atlas (TCGA) database in 2006. The TCGA is a human cancer gene information database established and collaborated by the National Cancer Institute (NCI) and the National Human Genome Research Institute (NHGRI), owning comprehensive and well-curated genomic data of over 11,000 tumors across 33 major cancer types. This database mainly includes the repository containing genomic, transcriptomic, epigenetic, proteomics, and clinical information of various kinds of cancer [11].

We combined the RNA sequencing (RNA-Seq) data and clinical information of AML patients downloaded from TCGA database to perform the present study. By screen and analyzing the DEGs related to prognosis of AML patients, we tended to identify some novel AML-related genes. For further determination of the association between their aberrant expression levels and prognosis, the nomogram prognostic model was constructed to predict the long-term survival rate of AML patients. Moreover, we enriched DEGs-related signaling pathways in different cytogenetic groups using gene ontology (GO) enrichment analysis. Finally, 87 Chinese adult AML patients and 43 children AML patients were recruited to validate the clinical value for diagnosis of our findings.

## Materials and methods

### Samples and data preprocessing

The RNA-Seq data and clinical information of TCGA-LAML were downloaded from the TCGA database (https://portal.gdc.cancer.gov/), and the raw data included 151 RNA-Seq samples for 60,488 genes and 200 clinical samples. Firstly, we filtered the genes with low expression (counts per million (CPM) < 1 in all samples), which reduced our RNA-Seq data to 56,822 genes. After excluding Asian race (2 cases), missing clinical information (2 cases), and missing cytogenetics risk information (3 cases), 193 clinical samples were left. For matching the information from different databases, only 146 samples were selected because of the explicit intersection of RNA-Seq and Clinical data. Considering 9 patients were excluded because of the lack of follow-up information, we selected only 137 samples for the further investigation. On the other hand of genes filtration, RNA-Seq data were reduced to 37,362 genes after removing genes with null expression in over 50% samples. We also excluded the genes with their high or low z-score covering 95% samples, and the RNA-Seq data decreased from 37,297 to 16,226 genes. Finally, we merged RNA-Seq data with clinical information, then obtained 137 samples with 16,226 genes expression profiles for final analysis.

### Screen differentially expressed genes

Cox proportional hazards model was used to evaluate the effects of gene expression level on the survival time of the patients with AML (*P* < 0.05, exp (coef) > 1). The DESeq2 R package (v3.5.1) was introduced to perform differential gene expression analysis between the low-risk and high-risk cytogenetics groups. The enrolling conditions for the differentially expressed gene were log_2_ Fold Change > 1 and adjusted *P* < 0.05. The Gene Set Enrichment Analysis (GSEA) of gene functional was performed by using ClusterProfiler R package, and the meaningfully enriched pathways were screened with abs (NES) ≥ 1 (abs refers to the absolute value), NOM *P* ≤ 0.05, and FDR *q* ≤ 0.25 as threshold. Venn diagrams were performed by using the limma R package to determine the intersection of survival-related DEGs, cytogenetics risk-related DEGs, and the genes in the top 10 pathways of up-regulation. By the result of Venn diagram, we further screened out the key genes of AML as our target for the present investigation.

### Survival analysis of DEGs

Multivariate Cox regression analyses were performed to analyze these intersectional genes as independent factors. Proportional hazards assumption was evaluated to test Cox regression model by Schoenfeld residuals. The Kaplan–Meier survival curve and Nomogram were plotted to predict and compare the 1-year, 3-year, and 5-year survival rates of AML patients. The concordance index (C-index) and calibration curve were used to evaluate the prediction performance of nomogram. All statistical tests were two sided and* P* < 0.05 was considered to indicate a statistically significant difference.

## Results

### Samples clinical characteristics

In the present study, a total of 137 AML patients were included in this analysis, including 77 males and 60 females, 126 whites and 11 blacks, with mean age of diagnosis as 54.44 ± 15.85 years. Using current SWOG criteria for cytogenetics risk category, the sample can be divided roughly into three categories: 28 patient had favorable cytogenetics, 74 had intermediate-risk/normal cytogenetics, and 35 had poor cytogenetics, 48 cases survival and 89 cases death. The clinical characteristics of included AML patients are shown in Table 1.

**Table 1:** Clinical characteristics of included AML patients in TCGA-LAML

Clinical character	Total (*n* = 146)	Percentage
Mean age at diagnosis (mean ± SD)	54.44 ± 15.85	
Gender		
Males	82	56.16
Females	64	43.84
Race		
Whites	135	
Blacks	13	
Cytogenetics risk category		
Favorable	30	20.55
Intermediate/Normal	80	54.79
Poor	36	24.66
Status		
Survival	53	36.30
Death	93	63.70

*SD* standard deviation

### Identification and analysis of survival- and cytogenetics-dual related DEGs

For survival-related DEG analysis, the data were adjusted for gender (male, female (reference group)), age, and race (white, Black or African American (reference group)). After screening by Cox proportional hazard regression analyses (*P* < 0.05, exp (coef) > 1), 1022 survival-related DEGs were obtained. 17 genes were lost by conversion of gene names, and finally 1005 survival-related DEGs were obtained.

**Fig. 1:**
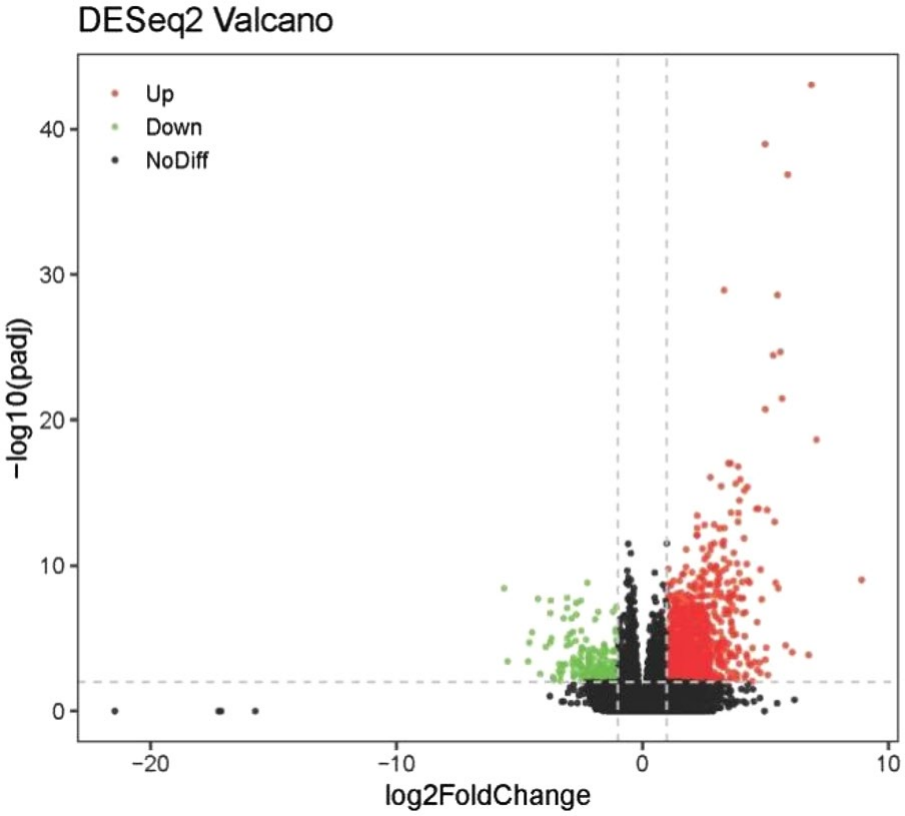
Volcano plots of category-related DEGs among LAML. Abscissa: variation in gene expression between different samples. Ordinate: the significance cytogenetics risk of the expression differences. Red dots represent up-regulated genes (log2 Fold Change > 1, *P* < 0.05). Green dots show down-regulated genes (log2 Fold Change < 1, *P* < 0.05). Black dots are genes with no significant difference

Considering the cytogenetics risk as an imperative clinical factor in AML**,** we further re-filtered the survival-related DEGs in different cytogenetics risks in order to find out the target genes which are also associated with AML cytogenetics risk. The cytogenetics risk category usually can be divided into two groups: favorable intermediate/normal risk (104 cases) and poor risk (33 cases). Genes were considered up-regulated (down-regulated) if log_2_ Fold Change in expression was higher (or lower) than 1 (abscissa), and adjusted *P* < 0.05 (ordinate), a total of 2291 cytogenetics risk category-related DEGs were identified using DESeq2 (as shown in Fig. 1).

**Fig. 2:**
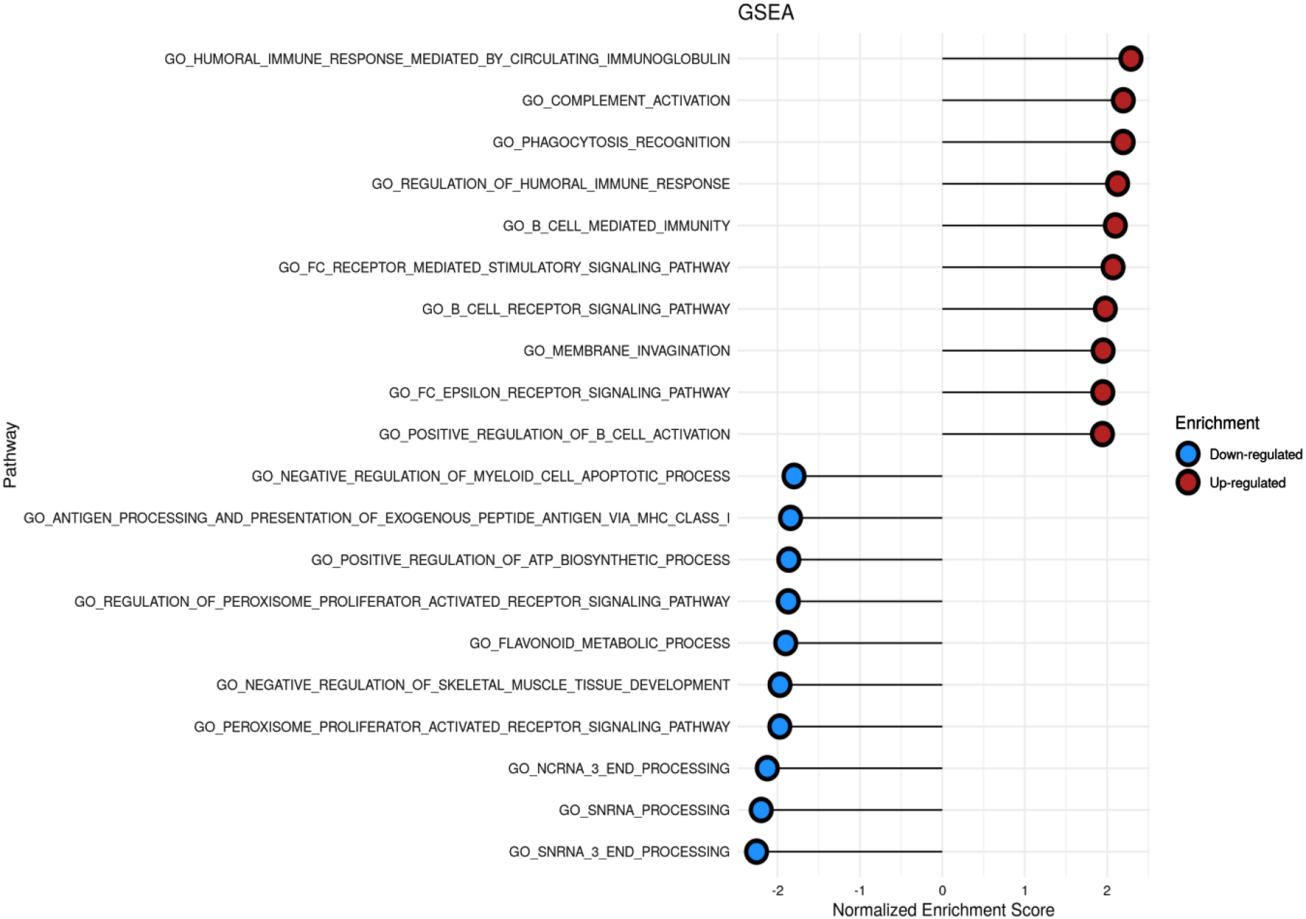
Top10 significant pathways of cytogenetics risk category among LAML by GSEA

Because the large number of survival-related DEGs might cost a huge calculation power to analysis, we performed the functional enrichment analysis to enrich the genes in DESeq2 with high relevancy biological functions. A total of 790 meaningful pathways of cytogenetics risk category were listed by GSEA. The top 10 pathways of up-regulated and down-regulated gene sets of NES are marked and listed in Fig. 2.

**Fig. 3:**
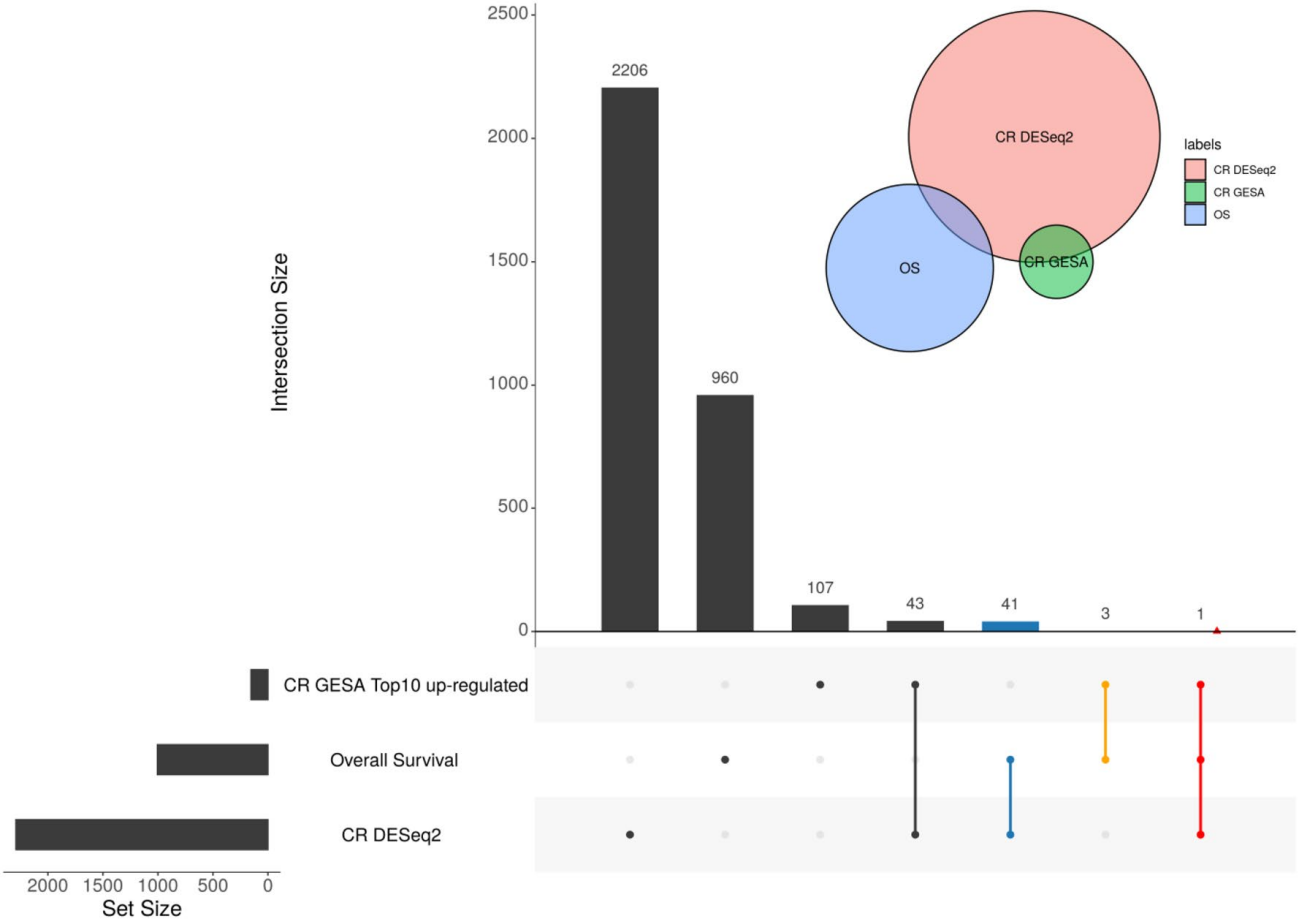
Venn diagram. CR GSEA Top10 up-regulated: Top10 significant pathways gene sets of cytogenetics risk category among LAML by GSEA. Overall Survival: gene sets whose expressions were significantly associated with survival rate. CR DESeq2: gene sets whose expressions were significantly associated with cytogenetics risk category

Finally, a Venn diagram was drawn to reflect the intersection of survival-related DEGs (Overall Survival), cytogenetics risk-related DEGs (CR DESeq2), and functional enrichment analysis (Fig. 3). A total of 42 intersecting DEGs were identified in the two sets, and there is only one gene in the CR GSEA top10 pathways of up-regulated among the 42 intersecting DEGs, namely, IGHM.

### Survival analysis of IGHM

**Fig. 4:**
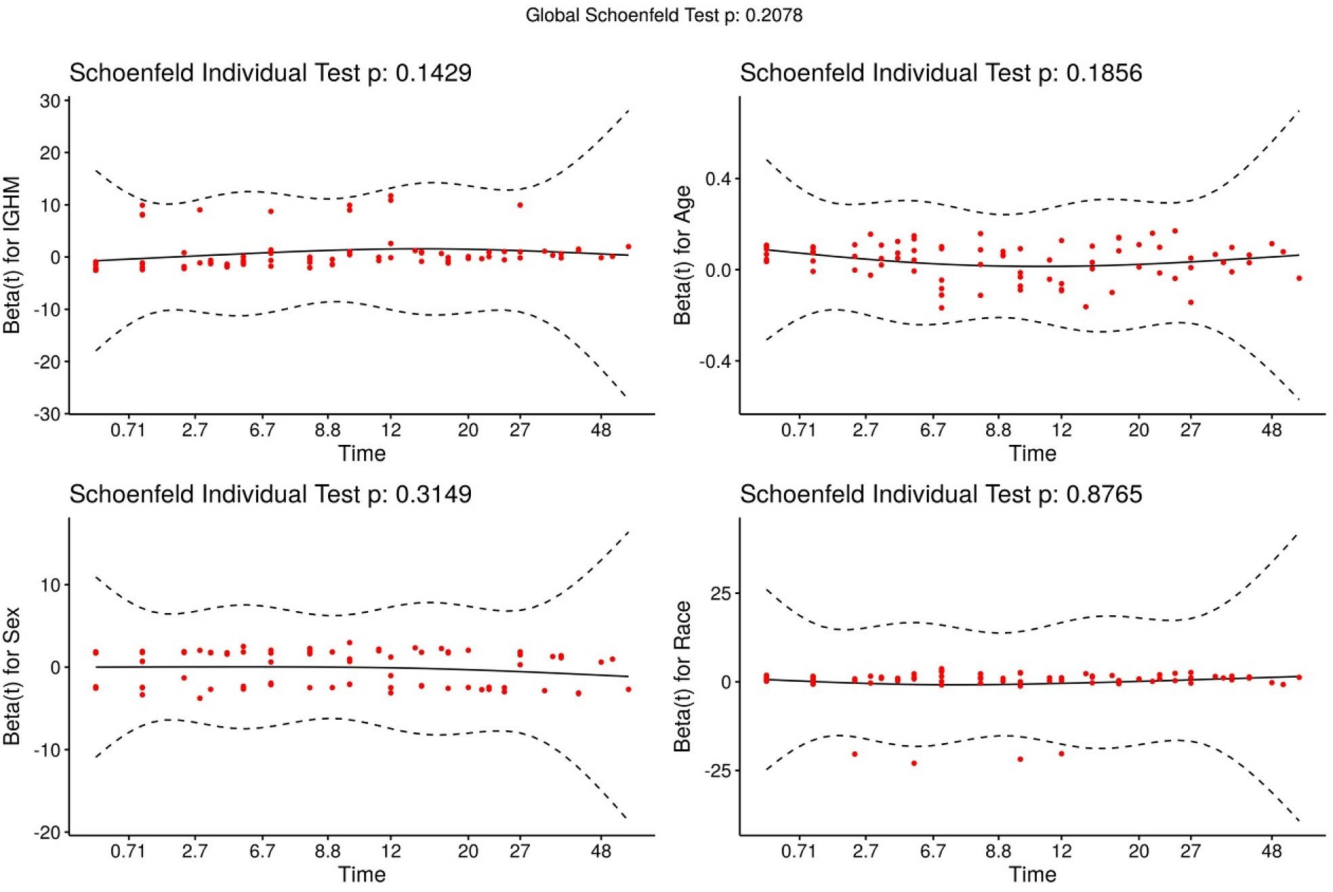
PH test for Cox model

Multivariate Cox regression analysis was used to analyze the influence of clinical characteristics and IGHM gene expression on the survival time of AML patients. Age, gender, and race were included as covariates, and the survival rate of patients with different IGHM amounts of expression was analyzed by Cox proportional hazards model. The model results of hypothesis testing show *P* = 0.21 (Fig. 4). The Wald test value of the overall Cox regression model is 31.54, and its *P* < 0.001. The average survival time of patients based on all factors was 19 months (95%CI 12 ~ 27 months), which is about 1.5 years. The results of multivariate analysis showed that the two factors, IGHM expression and age, have their statistical significance with the survival time of AML patients, and all *P*-values were  < 0.05 (Table 2).

**Table 2:** Multivariate Cox regression analysis of survival time in LAML patients

Factor	coef	Exp(coef) [95%CI]	Se(coef)	*Z*	*P*-value
IGHM	0.73	2.07 [1.03, 4.15]	0.36	2.04	0.041
Age	0.04	1.04 [1.02, 1.06]	0.01	4.74	< 0.001
Sex	−0.15	0.86 [0.55, 1.34]	0.23	−0.68	0.499
Race	−0.09	0.91 [0.33, 2.55]	0.52	−0.17	0.863

**Fig. 5:**
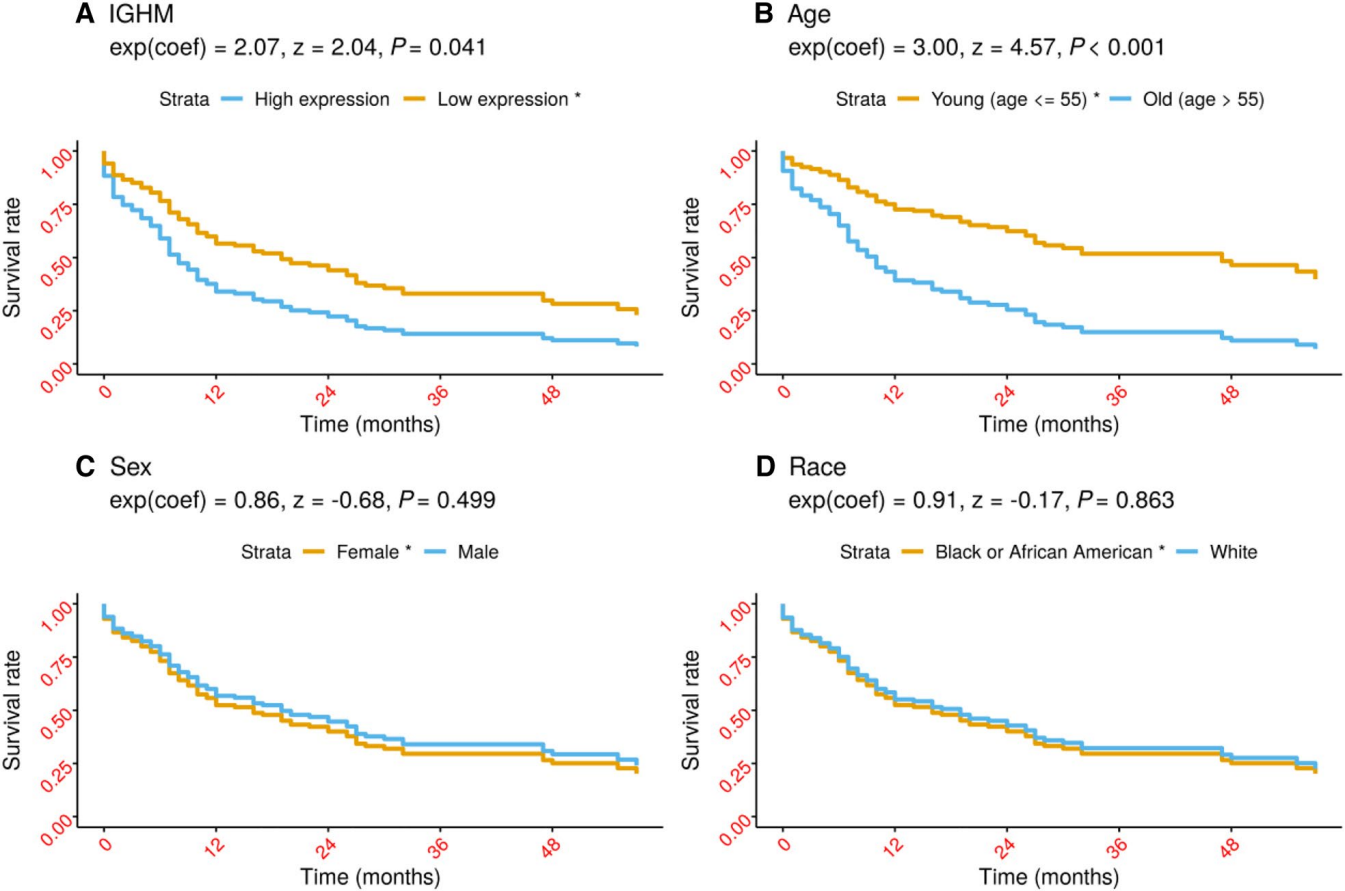
Adjusted survival curves for LAML survival. *: reference group

After adjusting for other variables, the Kaplan–Meier survival curve for each factor in different conditions is demonstrated in Fig. 5. The survival rate of patients in the IGHM high expression group was statistically lower than that in low expression group (*P* = 0.041). The risk of death in IGHM high expression patients was 2.07 times higher than that in low expression ones (*P* < 0.05). The mean value 55 years of age was used as a cut-off point because age is a continuous variable, these variables were converted into categorical variables by the cut function, and the survival curve was plotted. Kaplan–Meier survival analysis further showed that the survival rate of old group is lower than that of young group, and the difference was statistically significant (*P* < 0.001). The risk of death in the patients with old group having 3.00 times the risk compared to young patients (*P* < 0.001).

### Establish and evaluate nomogram prognostic model

**Fig. 6:**
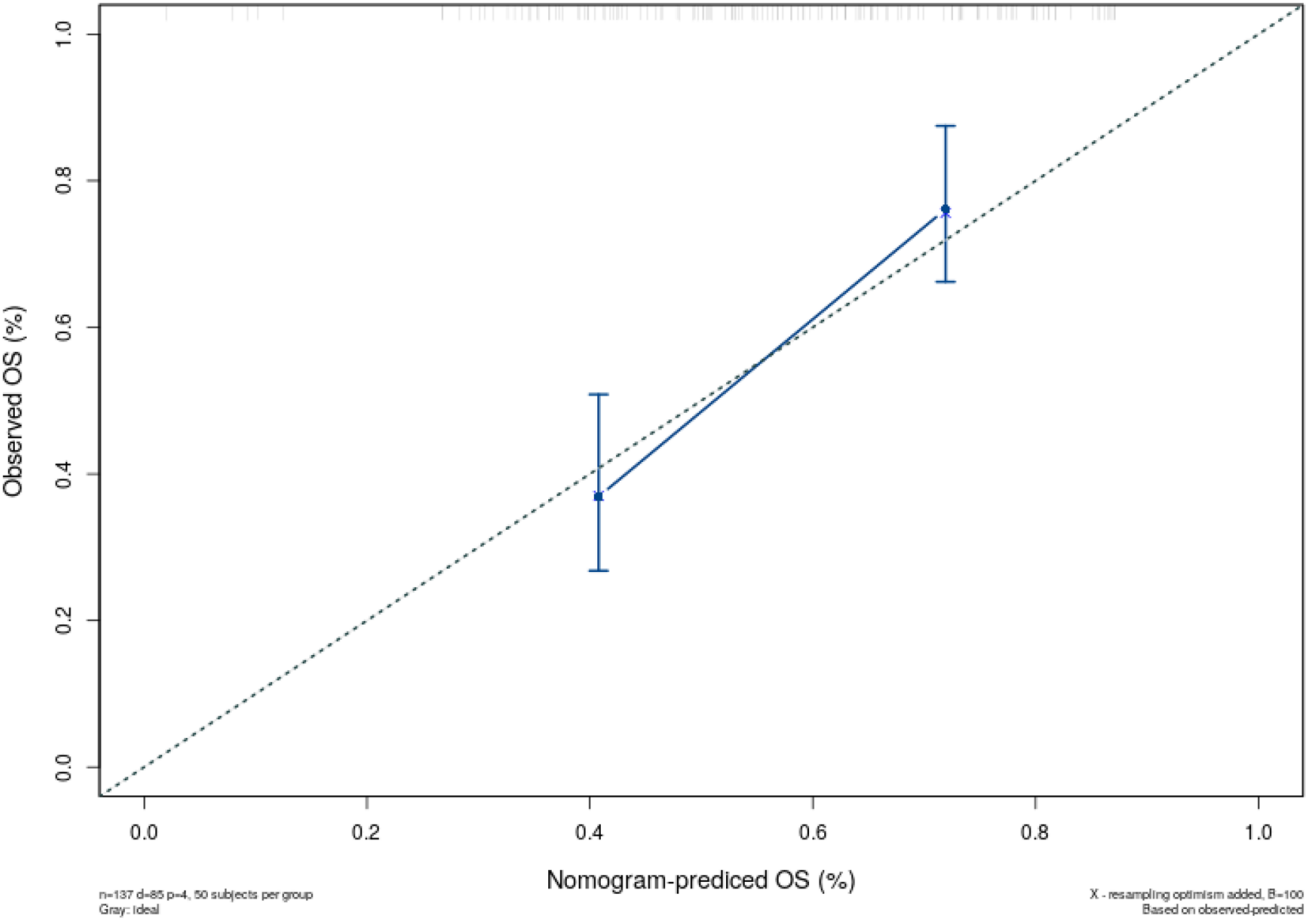
Calibration curve

we commonly use nomograms, which is also called alignment diagram, to estimate the survival of individual patient by incorporating multiple clinical variables and their interdependent relationships in medical research [12]. Its essence is the visualization of the built model, the closer the C-index of the nomogram is to 1, the better the accuracy of the model. In this study, the C-index of fitting the prediction model of Cox regression is 0.69, and this result shows that the model has a good accuracy. The calibration curve is shown in Fig. 6, and the predicted calibration curve is closer to the standard curve, and it also shows that the prediction ability of nomogram is better.

**Fig. 7:**
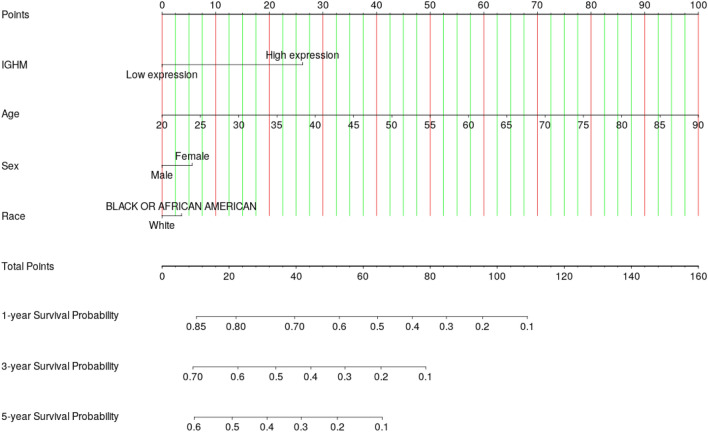
Nomogram fitting the cox proportional risk model

Nomogram results showed that the expression of IGHM in LAML patients has a great influence on their survival time. It is predicted that the 1-year, 3-year, and 5-year survival of the patients with low expression group of IGHM are  > 85%,  > 70%, and  > 60%, and the 1-, 3-, and 5-year survival of patients in the high expression group are predicted to be 68%, 43%, and 30%,respectively (in Fig. 7).

### Analysis of IGHM-related signaling pathways

After focusing IGHM as our target gene, we reviewed the results of GSEA analysis to evaluate the potential function of IGHM (in Table 3). Among the significant top 10 up-regulated pathways with statistical significance (abs (NES) ≥ 1, NOM *P* ≤ 0.05 and FDR *q* ≤ 0.25), IGHM participated in 7 pathways, including humoral immune response by circulating immunoglobulin, complement activation, phagocytosis recognition, B cell mediated immunity, B cell receptor signaling pathway, membrane invagination, and positive regulation of B cell activation.

**Table 3:** Significant top up-regulated pathways participated by IGHM

Pathway	ES	NES	NOM *p*-val	FDR *q*-val	Size
1. GO_HUMORAL_IMMUNE_RESPONSE_MEDIATED_BY_CIRCULATING_IMMUNOGLOBULIN	0.75	2.29	< 0.001	< 0.01	138
2. GO_COMPLEMENT_ACTIVATION	0.71	2.2	< 0.001	< 0.01	157
3. GO_PHAGOCYTOSIS_RECOGNITION	0.76	2.19	< 0.001	< 0.01	72
4. GO_B_CELL_MEDIATED_IMMUNITY	0.67	2.1	< 0.001	< 0.01	206
5. GO_B_CELL_RECEPTOR_SIGNALING_PATHWAY	0.66	1.98	< 0.001	< 0.01	112
6. GO_MEMBRANE_INVAGINATION	0.64	1.95	< 0.001	< 0.01	123
7. GO_POSITIVE_REGULATION_OF_B_CELL_ACTIVATION	0.64	1.94	< 0.001	< 0.01	131

*NES* normalized enrichment score, *NOM* nominal, *FDR* false discovery rate

Gene sets with abs (NES) ≥ 1, NOM *p*-val ≤ 0.05, and FDR *q*-val ≤ 0.25 were considered as significant

## Discussion

The present study analyzed the RNA-seq data and clinical information of 146 AML patients from TCGA database through bioinformatics analysis. In this study, we performed multivariate Cox regression analysis on prognostic factors, and the findings suggested that age and IGHM expression are independent prognostic factors for patients with AML (*P* < 0.05), which means IGHM could be a key gene which is statistically related to the AML patients’ survival. To our knowledge, it was the first study to confirm IGHM as a potential target gene to AML by using bioinformatics analysis, accompanied with further clinical sample verification.

Previous studies has shown that IGHM (Immunoglobulin Heavy Constant Mu) is a gene marker for the short transition period in the differentiation and development of myeloid and lymphoid progenitor cells [13, 14]. Normally, IGHM maintained in an inactive status, without antibody response. But in both B lymphocytic leukemia and myeloid leukemia, with the process of the cellular malignant transformation and abnormal clone amplification, the expression of IGHM was abnormally highly increased [15]. In our study, the high expression of IGHM was identified as an independent risky factor for AML patients’ survival, which was highly consistent with the conclusion that high expressions of IGHM are more common in AML patients [15, 16]. For the function of IGHM in coding protein, the IGHM defines the IgM isotype in B cells; aberrant expression of IGHM is closely associated with the misfunction of mu-chain of immunoglobulin, including mutations and rearrangements. A resent research suggests that patients with autosomal recessive agammaglobulinemic have IGHM gene mutations [17]. Also, the mu-chain of immunoglobulin gene rearrangement was detected in myeloid leukemia cells from AML patients, and the survival rate of AML patients was significantly lower [13, 14, 16, 18, 19]. Neeraj et al. also found the gene rearrangement of IGHM in the diffuse large B cell lymphoma (DLBCL) [20]. Therefore, we believed the IGHM could be a functional and reasonable marker to evaluate the prognosis of AML.

The present study also analyzes differences between the expressions of IGHM in different cytogenetic risk groups, and the results show that the expression of IGHM is higher in patients with high-risk group. Unlike most other solid tumors, many hematological malignancies strongly associated with single characteristic cytogenetic abnormalities. A previous study had shown that patients, younger than 65 years treated with standard chemotherapy with a favorable karyotype, have CR rates in the 85–90% range and a 5-year OS of 50–60% [21]. Also, patients with intermediate-risk cytogenetics have CR rates of 65–75% and a 5-year OS of 35–45%, while patients with poor cytogenetics have CR rates of 45–55% and a 5-year OS of only 10–20% [21]. Therefore, the problem of AML with unfavorable-risk cytogenetics deserves special attention. The GSEA of our study showed that DEGs mainly enriched in immune response (BP) regulated the occurrence and development of AML and influence the prognosis of AML, which was consistent with the contributes of immune response to tumor progression and drug resistance in various cancers [22, 23], including lung cancer [24], breast cancer [25], and bladder cancer [26]. We also found that IGHM genes were primarily enriched in the B cell-mediated immunity and the B cell receptor signaling pathway, and these results indicate that the development and prognosis of AML may be related to these biological processes.

There are several limitations existing in our study. Although the TCGA database contains information about Asians, blacks, whites, and other multi-ethnic groups, it still focuses on whites, and Asians can be included in the later for further verification. Moreover, this study concerns only the TCGA database, and only the RNA-seq data and clinical information, and the sample size is small, and we hope that future research can combine multiple databases and include data on DNA sequencing, methylation profiling, exome sequencing, and miRNA expression, in order to explore more molecular markers of the pathogenesis of AML. Finally, we did not check the status of mu-chain of immunoglobulin in our AML sample to evaluate the relationship between IGHM high expression and the rearrangement, because of the rare bone marrow samples of AML patients. Further investigations are expected to reveal the mechanism of how high IGHM expression leads to poor prognosis of AML.

In conclusion, we confirmed that IGHM is independent factor for the prognosis in AML patients. Besides, we also performed a nomogram model for predicting the long-term survival rate of AML patients, patients with high expression of IGHM showed lower survival, and the expression, in order to explore more molecular markers of the pathogenesis of AML. The further molecular biology experiments and clinical studies are needed to verify the possibility of IGHM as a prognostic molecular marker for AML.

## Data Availability

The datasets generated during the current study are available in the TCGA repository.
